# The challenges of epidemiologic translation: communicating with physicians, policymakers, and the public

**DOI:** 10.3389/fpubh.2024.1270586

**Published:** 2024-01-24

**Authors:** Jeff Levin

**Affiliations:** ^1^Institute for Studies of Religion and Medical Humanities Program, Baylor University, Waco, TX, United States; ^2^Department of Psychiatry and Behavioral Sciences, School of Medicine, Duke University, Durham, NC, United States

**Keywords:** epidemiology, translational medicine, health, communication, policy, ethics

## Abstract

Translational epidemiology refers to the practical application of population-health research findings to efforts addressing health disparities and other public health issues. A principal focus of epidemiologic translation is on the communication of results to constituencies who can best make use of this information to effect positive health-related change. Indeed, it is contended that findings from epidemiologic research are of greatest use only if adequately communicated to health professionals, legislators and policymakers, and the public. This paper details the challenges faced by efforts to communicate findings to the these constituencies, especially three types of miscommunication that can derail efforts at translation. These include perceived misinformation, perceived disinformation, and perceived censorship. Epidemiologists are ethically obliged to avoid these types of miscommunication, and, accordingly, are advised to place greater emphasis on messaging and media outreach to physicians, government officials, medical educators, and the general public.

## 1 Introduction

All epidemiologists, presumably, hope that their research makes a difference in the world, in the lives of populations at risk for the diseases that they investigate. Communicating findings to audiences outside of one’s professional community in order to ensure application to real-world public health issues, whether ongoing challenges or immediate threats, is vital to the task of epidemiologic translation (1). Just as scientists speak of translational research and physicians speak of translational medicine, so, too, has translational epidemiology become a topic for thoughtful if niched commentary within public health. Epidemiology is “at the epicenter of translational science” (2, p. 525), uniquely positioned among biomedical disciplines to focus on population-health research from problem formulation to application of findings to public health. With translational medicine now established within academic medicine and the U.S. National Institutes of Health (NIH) (3, 4), translation is poised to gain a higher profile within epidemiology. For this to happen, especially important are setting agendas for translation, identifying challenges and how to overcome them, and specifying consequences of failing to do so.

A key component of translational epidemiology is communicating findings to constituencies outside of epidemiology and academic public health. Failure is not merely a lost opportunity, but an ethical breech. Public health messaging is an “indispensable component” of any “robust” system of response to new epidemiologic information (5, p. 1). In this paper, the importance and implications of translation and associated challenges are summarized in order to encourage thoughtful attention among epidemiologists. The takeaway point is simple: *findings from basic research on population health are of greatest use only if adequately communicated to health professionals, legislators and policymakers, and the public*. This conclusion should be obvious—after all, who wishes to publish findings that no one sees and that never get applied to public health policy or intervention? Yet sometimes attending to translation gets subordinated to more logistical concerns related to conducting research. This is understandable in light of the multitasking involved but, still, is consequential and unfortunate.

## 2 Translational science

Translational epidemiology emerged from translational medicine which, in turn, evolved from earlier discussions of translation in science generally. In a sense, these concepts—translational research, translational medicine, translational epidemiology—are a nested series.

*Translational research*, first widely spoken of in the mid 1980s (6), has been subject to considerable writing, accounting for over 2 million hits on Google Scholar. It entails application of scientific discoveries to producing scientific or technical knowledge, solving scientific or technical problems, and bringing solutions to market (7). This concept is referenced in relation to numerous scientific disciplines and fields, as well as in engineering, education, the social sciences, and elsewhere.

The phrase *translational medicine* originated in the 1990s (8), subsequently producing over 67,000 hits on PubMed. Usage is expanding quickly: in 2022, the number was about 57,000. The translational function here bridges preclinical (i.e., basic, biomedical, bench) research and clinical (e.g., diagnostic, prophylactic, therapeutic) applications. Translational medicine is defined as applying research “from bench to bedside”—i.e., from the laboratory to clinical practice (9). A notable example is translation of basic vaccine research on variola in the 1970s (10) to later work by public health scientists who developed strategies to eradicate smallpox by the 1980s (11). For this particular effort, bench-to-bedside was more accurately bench-to-village, but the same principle held: basic biomedical research applied to a real-world globally-impacting medical issue.

First used around 2010 (12), *translational epidemiology* currently accounts for over 1,600 hits on PubMed. Similar in meaning to translational medicine, translation here is from epidemiologic findings to myriad public health functions for purposes of enhancing population health. These include identifying risk or protective factors and primary preventive strategies for chronic and acute diseases; contributing to disease surveillance and maintenance of vital statistics; planning behavioral interventions and other health promotion and disease prevention programs; health services planning and policymaking; environmental health activism; and developing medical treatments based on population-based medical outcomes research. Examples of studies which speak of epidemiologic translation, in those words, can be found for cancer epidemiology (13), psychiatric epidemiology (14), genomic epidemiology (15), even the epidemiology of religion (16).

## 3 Translating epidemiology

Numerous definitions of epidemiology exist, variously worded, a representative example being from the authoritative *A Dictionary of Epidemiology*: “The study of the occurrence and distribution of health-related events, states, and processes in specified populations, including the study of the determinants influencing such processes, and the application of this knowledge to control relevant health problems” (17, p. 95). Deconstructing this definition, “distribution” speaks to descriptive epidemiology: i.e., how much of a respective outcome is present, by categories of person, place, and time; “determinants” is about analytic epidemiology: i.e., what are a given outcome’s causes or antecedents or predictors; and “application” refers to applied epidemiology: i.e., how this information is used to address a particular public health issue, or, more specifically, to “promote, protect, and restore health” (17, p. 95). In defining epidemiology, the first two parts of this definition are universally acknowledged; applied epidemiology is often overlooked (18). This is where epidemiologic translation comes in, and is largely about communication. Specifically, “a critical component of translational epidemiology is communication and partnership with other stakeholders in the broader enterprise of evidence-based public health” (1, p. 2054).

In applying epidemiologic findings, translation is not just about developing new treatments, interventions, or policies, but also about communicating findings to constituencies who can make use of them to contribute to population health (19, 20). These include physicians, policymakers, and the public—respectively, the front-line workers, decision-makers, and general population. For each group, communication for purposes of translation brings associated challenges, practical and ethical, for which respective recommendations are offered.

In *communicating with physicians*, a principal challenge is conveying findings in ways that make biological sense, yet are relevant to medical doctors (and other health professionals). While not always obvious to epidemiologists, epidemiologic findings may be caveated in ways counterintuitive to clinicians, who may not be properly trained in interpreting epidemiologic findings (21). For example, epidemiologists describe exposure-outcome relationships that exist on average, across populations, and *caeteris paribus* (all things being equal) (22), yet, for novel results, epidemiologic findings may not be consistent with current understandings of pathophysiology nor provide useful etiologic information or therapeutic guidance. A suggestion is to seek collaboration with physicians and/or biomedical scientists or at least, where possible, to spell out any clinical implications in terms of diagnosis, treatment, or prevention.

In *communicating with policymakers*, an immediate challenge is disseminating information to individuals who formulate health policies and laws and fund public health interventions (23). Another challenge is that evidence favored by policymakers (e.g., systematic reviews, non-research data) may be unlike the findings produced by epidemiologists who conduct observational studies (24). There are also budgetary and political considerations. Gaining an audience with government decision-makers at the federal, state, regional, or local levels requires entrée to staffers and committees. Well-connected senior colleagues and administrators may be helpful. Also, academic epidemiologists should be assertive in media outreach. Every published study should be accompanied by a university press release, and one should accept most opportunities for media interviews, public presentations, and, if requested, testimony before legislative or regulatory bodies. If one’s study results go up against political, military, or industrial interests, one may be easily intimidated, but the advice here is simple if perhaps daunting: take a deep breath and restate one’s findings politely but unwaveringly.

In *communicating with the public*, the most critical initial challenge is transparency (25). Epidemiologists must be honest and forthright with the public, communicating as completely and truthfully as possible, in order to build “trust and credibility” (26, p. 245). Establishing media liaisons is critical, whether reporting on an outbreak, ongoing surveillance data, or study results. Of course, prematurely reporting information without confirmation does no one any favors, but so does failing to report accurate information or any information at all. The first few months of the COVID-19 pandemic in early 2020, in the U.S., U.K., and China, are a case study in how government and nongovernmental health agencies and officials who lose control of messaging can inadvertently create panic (27). Communicating epidemiologic findings to the public is complicated by difficulty in conveying the probabilistic nature of observed associations—results of observational studies are rightly phrased in the subjunctive tense, less likely using words like “cause” or “prove.” This presents challenges in translating, literally, population-health findings for public consumption through media channels looking for assertive headlines (28, 29).

## 4 When translation goes wrong

When full transparency is lacking or other challenges unmet, especially regarding public communication, the translational process can go awry. This is not necessarily the fault of epidemiologists; they may not have control over the messaging. This may be the responsibility of media people, if one is under the employ of a government agency, or of medical doctors or administrators who are changed with overseeing an investigation. However, there is a professional responsibility for epidemiologists to be aware of how public communication can go sideways, so to speak, and to strive to prevent this or mitigate its consequences.

Added to this are the realities of medical news reporting, whereby medical reporters face their own pressures which sometimes means that “[i]nformation is delivered rapidly, [with] little time … taken to provide a context for the story. Instead, the reporting is sensationalized: The journalist overstates a scientific finding and, as a result, the public is misled about the implications of that finding” (30, p. 976). The consequences can be serious, especially in an outbreak of a novel pathogen, a toxic mass exposure, or an epidemic of a serious condition hitherto hidden from view. Most epidemiologists could likely identify instances of miscommunication, not all of which are publicly known. For epidemiologic research, miscommunication can take various forms depending upon whether intentional or unintentional, including misinformation, disinformation, and censorship, or, rather, public perceptions of such.

A recent example of *perceived misinformation* involves the initial SARS-CoV-2 outbreak in 2019–20. Because global interinstitutional coordination was still lacking, contradictory information about pathophysiology, transmission, clinical course, prognosis, therapeutics, and prophylaxis proliferated. To deal with the concomitant uncertainty and fear, lay people increased their reliance on internet searches and social media coverage, which exacerbated the spread of misinformation (31). Global nongovernmental agencies such as the World Health Organization; national agencies such as the U.S. Centers for Disease Control and Prevention and the NIH; and the political leadership of many nations, including the U.S., the U.K., Russia, and China, issued conflicting statements and recommendations regarding most of the above, and guidelines continually changed (32), which made this situation worse.

Nothing nefarious is implied here; this was hardly unexpected given the abrupt onset and widespread scope of the pandemic. Moreover, this was hardly the fault of the epidemiologists on the ground who were laboring to monitor and investigate the outbreak. However, without coordinated messaging early on, the deleterious societal impact of the pandemic was accelerated and reinforced, spawning dangerous levels of COVID-19 skepticism and SARS-CoV-2 vaccine hesitancy (33). Correcting misinformation is not simple (34), yet without a single trustworthy source of news updates, such situations risk giving an appearance to the public that no one is charge or knows what they are talking about. Even if untrue and grossly unfair, this may become tacit public perception, causing the lay public to lose confidence in appointed experts and fuel conspiratorial thinking that something sinister is afoot. Sadly, this was observed during the pandemic, leading to “a dramatic infodemiological scenario” (35, p. 226). The reputation of the epidemiology profession seems to have suffered as a result, which is probably unmerited. Subsequent calls have been made for field epidemiologists to become more savvy in developing the requisite public communication skills to function in the current media environment during times of global health emergencies (36).

By contrast, examples of *perceived disinformation*, or deliberate or “fabricated” misinformation (37), are also legion. While not necessarily more serious or impactful than perceptions of misinformation, they can be more disheartening because of the possibility that a crisis might have been averted sooner or more effectively, or some of its consequences prevented, had the disinformation been interdicted. A recent example involves derailment of a Norfolk Southern freight train carrying multiple hazardous materials in East Palestine, Ohio, in 2023 (38). Public statements were made almost immediately by government agencies and representatives of the railroad and chemical industries indicating that the situation was under control and substantial health risks to residents were unlikely, despite widespread reports of symptomatology (39). These all-clear statements were made before a full investigation was completed; in fact, the wreckage and its toxic contents were not yet completely cleaned from the site.

Concerns about a coordinated cover-up and complaints about non-responsiveness from government and industry began circulating, including from environmental activist Erin Brockovich (40), but were dismissed by officials. This official reaction was perceived as cavalier and in turn heightened public distrust even further, creating a layer of conspiracy-driven misinformation on top of the existing miscommunication problems (41). There is little indication that government epidemiologists, while looped into deliberations, were the ultimate decisors on what public statements were issued; their job was to collect health data and report it up the chain. But as with the COVID-19 pandemic, poor communication—even if unintentional—exacerbated an emotionally and politically challenging situation, created barriers to effective mitigation, heightened resistance to subsequent official reports, and made an already serious crisis more intractable.

The final type of miscommunication is *perceived censorship*. Actual censorship is harder to document after the fact, by definition, since records or reports may have been suppressed or never written down. The censoring of scientific findings, however, is “commonplace in much government and corporate research” (42, p. 2167), including health-related research and especially when findings conflict with a protected political agenda. An example, which unfortunately cannot be proven for these reasons, is documentation—or, rather, non-documentation—by U.S. government scientists in the early 1990s of elevated rates of sexually transmitted diseases among child victims of parental incest. For purposes of the present paper, this reporting is considered hearsay, but is mentioned here based on personal communication from a trustworthy scientist employed by a federal agency at the time. For reasons unclear to this source, the agency did not want the information made public. Ironically, subsequent research from the academic sector, years later, backed up the finding (43) and the earlier (alleged) cover-up was forgotten. Again, not to belabor the point, but it is probably unfair to lay this political intrigue at the feet of the epidemiologists and other investigators who conducted the research that compiled this information. But, still, there is an implicit duty among epidemiologists to guarantee that the truth or reality of findings be communicated clearly, even if the findings themselves may be somewhat equivocal.

The type of heavy-handed institutional response noted above is not unprecedented. The possible association, for example, between exposure to extremely low-frequency electric and magnetic fields and subsequent childhood leukemia incidence, and the purported duplicity of power companies in covering this up (44), was debated for decades before a comprehensive review of empirical studies and meta-analyses cast doubt on the most strident claims and labeled the issue inconclusive (45). No matter, the subject remains disputed and highly charged (46). So a warning is in order, this time to media consumers and interpreters of population-health research findings: it may be tempting to claim censorship, but without conclusive proof this is a dangerous gambit, even though the confluence of powerful government and corporate interests may indeed work to conceal important epidemiologic findings.

To be fair, much of the time epidemiologists do a commendable job of public translation of their findings. Results are communicated clearly, succinctly, and truthfully, and the correct message reaches the public. Examples of exposure-outcome associations that have been well communicated and generally understood include the links between cigarette smoking and lung cancer (47), hypertension and stroke (48), and obesity and type 2 diabetes (49). Granted, what respective constituencies do with such information after the fact is out of the control of epidemiologists, which can be frustrating.

Where communication is successful, it has usually been the result of careful, comprehensive, and coordinated dissemination of the most accurate, up to date, and scientifically vetted information that is available. This is done through mechanisms such as consensus reports (50), federal guidelines (51), community prevention trials (52), social marketing campaigns (53), and updated content on board-certification exams (54). These approaches ensure that the latest findings reach both the medical and lay communities. Yet while such knowledge may be well diffused in the general population—everyone knows, for example, that smoking is bad for you—this has not ensured that lay people all make the wisest health-directed choices. But epidemiologists cannot reasonably be faulted for this; translation has been successful, even if adoption of recommended best practices is still lagging. Still, this suggests a need for better coordination among epidemiologists other public health specialists, including community health educators (55).

In other instances, communication of exposure-outcome associations was initially successful but the gains have been lost. For example, the message that immunization has been a historically effective tool for primary prevention of once highly incident childhood communicable diseases seems of late to be getting swamped by a wave of social media mis- and disinformation (56). In addition, knowledge of dietary and other risk factors for coronary artery disease has long been well dispersed though not entirely understood or acted on wisely (57). Despite a plethora of diet- and exercise-related information in the marketplace, much of it scientifically validated, the incidences of obesity and diabetes in the U.S. population continue to rise (58), alongside declining levels of cardiorespiratory fitness among youths (59). Again, epidemiologists may have done their part, but something is being missed. The job of epidemiologic translation, especially in a time of such rapid evolution in channels of mass communication, may not end with simply getting the word out. In the future, translation may entail and require a more ongoing approach. The typical epidemiologist may find oneself more often filling the role of a “public scientist” with its requisite willingness to participate in public engagement activities (60), something for which most in the profession may be unprepared, technically and psychologically.

Such a public role may be a considerable challenge, as a complicating factor is the observation that in communicating with public health policymakers, for example, epidemiologic researchers are confronted with the inherent conflicts and tensions between these two distinctive professional cultures, such as concerning the meaning and implications of concepts like risk, exposure, and confounding. Policymakers are all about promoting decisive action in the public sector; epidemiologists are focused on documenting what is oftentimes considerable nuance and ambiguity and then publishing these findings. It remains an ongoing challenge for epidemiologists to wade into the policy domain (61), but, as noted, this may need to become a role that more in the profession take on in the future.

## 5 Ethical obligations in translational epidemiology

To summarize, three questions are posed: Are there consequences to failing to adequately translate findings? Are there consequences to miscommunicating findings to intended audiences? Is it unethical for epidemiologists to disregard translation? The answer to each question is an unqualified “yes.” When findings are miscommunicated to physicians and other health professionals, to policymakers and public officials, and to the general population, the work of epidemiologic translation and evidence-based “knowledge translation,” more generally, may be derailed (62, 63). This is so whether reporting on a classic outbreak, an acute disease or accident scenario, or results of a large population study of an exposure or chronic disease. For the public health field, and for epidemiology in particular, moreover, this is also an ethical issue that requires acknowledgment.

Public health ethics, as a domain of theoretical concern and practical application, is something of a newcomer to the larger field of bioethics (64). Yet it has matured to the point of establishing normative understandings of the obligations of the public health sector to the general population. By now, there are many overlapping models or frameworks detailing these obligations (65). These include “general moral considerations” familiar to bioethics in general, such as beneficence, autonomy, and avoiding harm (66, pp. 171–172). But, in addition, there are considerations such as stewardship, trust, nondiscrimination, reciprocity, utility, accountability, equity, and respect for diversity (65), and still other obligations emphasizing the communal nature of public health, such as affirmation of solidarity (67). These obligations in turn derive from the widely acknowledged distinctives of the public health ethic: a focus on primary prevention, recognition of the multifactoriality of population-health risks, values grounded in communitarianism and social justice, and a global outlook (68). Accordingly, getting this issue right—that is, ensuring that accurate data on population health, including frightening and widespread outbreaks of emerging infections, are communicated to physicians, to policymakers, and to the public—is required not simply to meet an acceptable standard of professional practice but to fulfill a moral obligation that comes with the privilege of having trained to become an epidemiologist.

Consider the consequences off failing to fulfill this obligation. During the first few months of the COVID-19 pandemic, in 2020, preliminary information from population-wide surveillance and a plethora of meta-analyzed studies from around the world suggested elevated risk among certain groups, including older adults (although precisely which age cohorts was unclear), obese individuals (although the precise degree of overweight was unclear), and people with certain co-morbidities (although precisely which ones were unclear) (69). Further, it remained unclear whether this elevated risk could be observed for exposure, for infection, for caseness, for hospitalization, or for fatality, or some combination which itself perhaps varied across certain population groups. In short, the status of the emerging epidemiology of COVID-19 on the ground, one might say, was a mix of some very promising leads combined with a great deal of uncertainty. Yet this epidemiologic status report was not adequately conveyed to the public, both in the U.S. and abroad, as seen in the responses to this information. The valid demographic observations were either overstated or ignored, depending upon the government, medical, or media source; and the uncertainty was either downplayed or overhyped, again depending upon the source and its respective political agenda (70). This served to complicate the work of field epidemiologists and jeopardized perceptions of their competence and integrity (71).

As a result, the ethical charge incumbent on epidemiologists, noted above, was compromised. Those individuals and population groups most at risk were not always prioritized in primary-prevention strategies (heightening discrimination in providing care and services and harming efforts ay health equity) (72), accountability among government decision-makers was often absent (and just who these people were was often unclear, or it changed by the day) (73), and, aside from the harm this did to population health, in terms of morbidity and mortality, it also eroded public trust in the medical profession (74). Is this all or mainly the fault of epidemiologists? Certainly not. But the epidemiologic profession was at the front lines of gathering the data that informed—or failed to inform—a federal response that, at least in the U.S., has rightly been characterized as “disjointed, chaotic, and confusing” (75, p. 512).

But it is not just epidemiologists who are burdened with ethical obligations. Other constituencies, it could be argued, are obliged as well with responsibilities related to the communication of epidemiologic findings. For effective translation of such findings, specific concerns can be identified with accompanying ethical obligations on the part of various constituencies. First, taxes often pay for epidemiologic research; thus *government* is obliged to be transparent in communicating conflicts of interest and gaps in knowledge. Second, the health of families and communities may be at stake; thus *the public* is obliged not to be passive consumers of media spin about the latest studies. Third, the future of medical care is built upon research; thus *healthcare providers* are obliged to keep up with the published literature. Finally, medical, health professions, and pre-health education depend upon new knowledge; thus *educators* are obliged to train students to be independent and intelligent users of published research.

Despite each of these constituencies’ obligations, substantial barriers may inhibit their fulfillment. For example:

- Government, the public, healthcare providers, and medical educators, no matter how earnest, may meet resistance in discharging their ethical duties to communicate accurate health information. For example, industry representatives have long dominated government regulatory agencies (76), including those charged with oversight of public health functions. Thus, publicizing newly discovered environmental risk factors may be inhibited.
- The internet and social media have been colonized in no small part by uninformed or deceitful people who gather public followings and become opinion leaders regarding health and medical care (77). For example, hundreds of websites featuring self-appointed experts peddle unvalidated claims regarding wellness, diet, supplements, unusual therapies, and other sketchy health regimens and, more recently, especially during the COVID-19 pandemic, have created and reinforced widespread public skepticism about immunization. The medical internet has rightly been called a “quagmire” (78, p. 2295).
- Physicians and other clinicians find regulatory and reporting requirements and associated paperwork increasingly burdensome and time-consuming, and, as well, are losing their diagnostic and therapeutic autonomy (79). Thus, keeping up with the medical literature now must compete with ever increasing administrative obligations that many clinicians find overwhelming as they wrestle with professional burnout.
- Medical educators are confronted with too much scientific and clinical information (including spurious information) and too many information channels to be able keep up with every new finding, and they lack sufficient time and resources to train students to triage and vet it all. Further, medical and health professions students, in general, may not be formally or adequately trained to read and interpret research studies, a phenomenon long lamented by medical educators (80). Perhaps they get a lecture on this subject in an epidemiology course, but even this is not the norm.

In summary, to restate the takeaway point from earlier: *findings from basic research on population health are of greatest use only if adequately communicated to health professionals, legislators and policymakers, and the public*. This is not meant to suggest that the epidemiologic profession has failed at this—quite the contrary—but as a continued call to action. Epidemiology has a longstanding tradition of translation, if not always under that moniker. Ultimately, as Morabia noted, “History also gives us good reasons to be confident about the bright future of translational epidemiology” (81, p. 718). But there is an important caveat. This optimism depends upon whether epidemiologists are successful at disseminating research results, including the successes of applications of these results, to wider audiences outside of the profession and outside of academic public health (82). For this to happen, the future of epidemiologic training will need to account for the “changing health communication environment” (83, p. 462) and epidemiologists will need to become more adept at meeting the challenges of communicating findings accurately and dispassionately to the multiple constituencies that their research impacts.

## Data availability statement

The original contributions presented in the study are included in the article/supplementary material, further inquiries can be directed to the corresponding author.

## Author contributions

JL: Writing – original draft, Writing – review & editing.

## References

[1] Windle M Lee HD Cherng ST Lesko CR Hanrahan C Jackson JW. From epidemiologic knowledge to improved health: a vision for translational epidemiology. Am J Epidemiol. (2019) 188:2049–60. doi: 10.1093/aje/kwz085, PMID: 30927354 PMC8045479

[2] Hiatt RA. Invited commentary: the epicenter of translational science. Am J Epidemiol. (2010) 172:525–7. doi: 10.1093/aje/kwq212, PMID: 20688901

[3] Collins FS. Reengineering translational science: the time is right. Sci Transl Med. (2011) 3:90cm17. doi: 10.1126/scitranslmed.3002747, PMID: 21734173 PMC5101940

[4] Zerhouni EA. Translational and clinical science—time for a new vision. N Engl J Med. (2005) 353:1621–3. doi: 10.1056/NEJMsb053723 16221788

[5] Nan X Iles IA Yang B Ma Z. Public health messaging during the COVID-19 pandemic and beyond: lessons from communication science. Health Commun. (2022) 37:1–19. doi: 10.1080/10410236.2021.1994910 34724838

[6] Mellstedt H Schrijvers D Bafaloukos D Greil R. eds. ESMO handbook of principles of translational research. Abingdon: Informa Healthcare (2007).

[7] Curry SH. Translational science: past, present, and future. Bio Techniques. (2008) 44:ii–viii. doi: 10.2144/000112749 18422489

[8] Wehling M ed. Principles of translational science in medicine: From bench to bedside. 3rd ed. London: Academic Press (2022).

[9] Woolf SH. The meaning of translational research and why it matters. JAMA. (2008) 299:211–3. doi: 10.1001/jama.2007.26, PMID: 18182604

[10] Henderson DA. Smallpox: The death of a disease: The inside story of eradicating a worldwide killer. Guilford, CT: Prometheus Books (2009).

[11] Foege WH. House on fire: The fight to eradicate smallpox. Berkeley, CA; New York, NY: University of California Press, Milbank Memorial Fund (2011).

[12] Khoury MJ Gwinn M Ioannidis JPA. The emergence of translational epidemiology: from scientific discovery to population health impact. Am J Epidemiol. (2010) 172:517–24. doi: 10.1093/aje/kwq211, PMID: 20688899 PMC2927741

[13] Fu Z Zhang R Li P Jia M. Translational epidemiology: the powerful tool for precision cancer medicine. J Cancer Res Ther. (2019) 15:269–71. doi: 10.4103/jcrt.JCRT_276_18, PMID: 30964096

[14] Weissman MM Brown AS Talati A. Translational epidemiology in psychiatry: linking population to clinical and basic sciences. Arch Gen Psychiatry. (2011) 68:600–8. doi: 10.1001/archgenpsychiatry.2011.47 21646577 PMC3268346

[15] Boerwinkle E. Translational genomics is not a spectator sport: a call to action. Genet Epidemiol. (2012) 36:85–7. doi: 10.1002/gepi.21607, PMID: 22851471

[16] Levin J. Toward a translational epidemiology of religion: challenges and applications. Ann Epidemiol. (2022) 75:25–31. doi: 10.1016/j.annepidem.2022.08.053 36058543

[17] Porta M ed. A dictionary of epidemiology. 6th ed. Oxford: Oxford University Press (2014).

[18] Wynder EL. Applied epidemiology. Am J Epidemiol. (1985) 121:781–2. doi: 10.1093/oxfordjournals.aje.a114048 4014170

[19] Remington PL. Communicating epidemiologic information In: Brownson RC Petitti DB, editors. Applied epidemiology: Theory to practice. New York, NY: Oxford University Press (1998). 323–48. doi: 10.1093/oso/9780195111903.003.0011

[20] Ogilvie D Craig P Griffin S Macintyre S Wareham NJ. A translational framework for public health research. BMC Public Health. (2009) 9:116. doi: 10.1186/1471-2458-9-116, PMID: 19400941 PMC2681470

[21] Estellat C Faisy C Colombet I Chatellier G Burnand B Durieux P. French academic physicians had a poor knowledge of terms used in clinical epidemiology. J Clin Epidemiol. (2006) 59:1009–14. doi: 10.1016/j.jclinepi.2006.03.005, PMID: 16895826

[22] Levin J. Alex Broadbent. Philosophy of epidemiology [book review]. Theor Med Bioeth. (2014) 35:311–4. doi: 10.1007/s11017-013-9274-0

[23] Hennink M Stephenson R. Using research to inform health policy: barriers and strategies in developing countries. J Health Commun. (2005) 10:163–80. doi: 10.1080/10810730590915128, PMID: 15804906

[24] O’Donoughue Jenkins L Kelly PM Cherbuin N Antsey KJ. Evaluating and using observational evidence: the contrasting views of policy makers and epidemiologists. Front Public Health. (2016) 4:267. doi: 10.3389/fpubh.2016.00267, PMID: 27999772 PMC5138237

[25] O’Malley P Rainford J Thompson A. Transparency during public health emergencies: from rhetoric to reality. Bull World Health Organ. (2009) 87:614–8. doi: 10.2471/BLT.08.056689, PMID: 19705012 PMC2733257

[26] Tumpey AJ Daigle D Nowak G. Communicating during an outbreak or public health investigation. In: Rasmussen SA Goodman RA, editors. The CDC field epidemiology manual. New York, NY: Oxford University Press (2019). 243–59.

[27] Garrett L. COVID-19: the medium is the message. Lancet. (2020) 395:942–3. doi: 10.1016/S0140-6736(20)30600-0 32171075 PMC7154501

[28] Session on Public Engagement and Translation. Abstracts from the 20th IEA World Congress of Epidemiology, Anchorage, Alaska, USA, 17–21 august 2014 WCE2014. Int J Epidemiol. (2015) 44 (suppl. 1):i51–2.

[29] Smith GD. How do we know, what do we know and what can knowledge do?: from John Brownlee to translational medicine. Int J Epidemiol. (2008) 37:911–3. doi: 10.1093/ije/dyn210, PMID: 18839447

[30] Shuchman M Wilkes MS. Medical scientists and health news reporting: a case of miscommunication. Ann Intern Med. (1997) 126:976–82. doi: 10.7326/0003-4819-126-12-199706150-00008, PMID: 9182476

[31] Baerg L Bruchmann K. COVID-19 information overload: intolerance of uncertainty moderates the relationship between frequency of internet searching and fear of COVID-19. Acta Psychol. (2022) 224:103534. doi: 10.1016/j.actpsy.2022.103534, PMID: 35189539 PMC8843333

[32] Baker SA. Tackling misinformation and disinformation in the context of C-19. Cabinet Office C19 Seminar Series (2020). Available at: https://openaccess.city.ac.uk/id/eprint/24612/8/Think_piece_Stephanie_Baker_v1.pdf (Accessed August 1, 2023).

[33] Levin J Bradshaw M. Determinants of COVID-19 skepticism and SARS-CoV-2 vaccine hesitancy: findings from a national population survey of U.S. adults. BMC Public Health. (2022) 22:1047. doi: 10.1186/s12889-022-13477-2 35614396 PMC9132354

[34] van der Meer TGLA Jin Y. Seeking formula for misinformation treatment in public health crises: the effects of corrective information type and source. Health Commun. (2019) 35:560–75. doi: 10.1080/10410236.2019.1573295, PMID: 30761917

[35] Rovetta A. Health communication is an epidemiological determinant: public health implications for COVID-19 and future crises management. Health Promot Perspect. (2022) 12:226–8. doi: 10.34172/hpp.2022.28, PMID: 36686052 PMC9808906

[36] Hahné S Hammer C Tostmann A Whelan J Williams C. Field epidemiology: fit for the future. Euro Surveill. (2023) 28:2300347. doi: 10.2807/1560-7917.ES.2023.28.36.2300347, PMID: 37676144 PMC10486192

[37] Lwin MO Lee SY Panchapakesan C Tando E. Mainstream news media’s role in public health communication during crises: assessment of coverage and correction of COVID-19 misinformation. Health Commun. (2023) 38:160–8. doi: 10.1080/10410236.2021.1937842 34157919

[38] Lenharo M. Ohio train derailment: scientists scan for lingering toxics: East Palestine residents look to independent researchers to fill gaps left by authorities. Nature. (2023). doi: 10.1038/d41586-023-00820-9 36941417

[39] Goodman B Alvarado C. East Palestine residents worry rashes, headaches and other symptoms may be tied to chemicals from train crash. CNN Health (2023). Available at: https://www.cnn.com/2023/02/17/health/ohio-derailment-rashes-health-impacts/index.html (Accessed August 1, 2023).

[40] Worrnell T. Brockovich calls response to derailment a “cover-up”. NewsNation (2023) (March 31). Available at: https://www.newsnationnow.com/us-news/midwest/ohio-train-derailment/erin-brockovich-east-palestine-train-derailment-cover-up/ (Accessed August 1, 2023).

[41] Jennemann A. Tackling false information after the East Palestine, Ohio train derailment. International Journalists’ Network (2023) (March 28). Available at: https://ijnet.org/en/story/tackling-false-information-after-east-palestine-ohio-train-derailment (Accessed August 1, 2023).

[42] Martin B. Science: contemporary censorship In: Jones D, editor. Censorship: A world encyclopedia, volume 4, s-z. London, UK; Chicago, IL: Fitzroy Dearborn Publishers (2001). 2167–70.

[43] Bechtel K. Sexual abuse and sexually transmitted infections in children and adolescents. Curr Opin Pediatr. (2010) 22:94–9. doi: 10.1097/MOP.0b013e32833502ad 19952927

[44] Taubes G. EMF-cancer links: yes, no, and maybe. Science. (1993) 262:649. doi: 10.1126/science.8235582 8235582

[45] Working IARC. Group on the evaluation of carcinogenic risks to humans. IARC Working Group on the Evaluation of Carcinogenic Risks to Humans. Non-ionizing radiation, part 1: static and extremely low-frequency (ELF) electric and magnetic fields. IARC Monogr Eval Carcinog Risks Hum. (2002) 80:1–395. 12071196 PMC5098132

[46] Brabant C Geerinck A Beaudart C Tirelli E Geuzaine C Bruyère O. Exposure to magnetic fields and childhood leukemia: a systematic review and meta-analysis of case-control and cohort studies. Rev Environ Health. (2022) 38:229–53. doi: 10.1515/reveh-2021-0112, PMID: 35302721

[47] Tas F Erturk K. Online public interest in smoking and lung cancer: a comparative study in Google trends. J Cancer Res Ther. (2023). doi: 10.4103/jcrt.jcrt_276_22 38384027

[48] Ahuja R Ayala C Tong X Wall HK Fang J. Public awareness of health-related risks from uncontrolled hypertension. Prev Chronic Dis. (2018) 15:E40. doi: 10.5888/pcd15.170362, PMID: 29625630 PMC5894300

[49] Kayyali R Slater N Sahi A Mepani D Lalji K Abdallah A. Type 2 diabetes: how informed are the general public? A cross-sectional study investigating disease awareness and barriers to communicating knowledge in high-risk populations in London. BMC Public Health. (2019) 19:138. doi: 10.1186/s12889-019-6460-7, PMID: 30704425 PMC6357431

[50] Giovannucci E Harlan DM Archer MC Bergenstal RM Gapstur SM Habel LA. Diabetes and cancer: a consensus report. CA Cancer J Clin. (2010) 60:207–21. doi: 10.3322/caac.20078 20554718

[51] Lean MEJ Han TS Seidell JC. Impairment of health and quality of life using new US federal guidelines for the identification of obesity. Arch Intern Med. (1999) 159:837–43. doi: 10.1001/archinte.159.8.837, PMID: 10219929

[52] Schooler C Farquhar JW Fortmann S Flora JA. Synthesis of findings and issues from community prevention trials. Ann Epidemiol. (1997) 7 (S7):S54–68. doi: 10.1016/S1047-2797(97)80008-7

[53] Colquhoun H Ellen M Brehaut J Weinreich NK Morvinski C Zarshenas S. Potential social marketing applications for knowledge translation in healthcare: a scoping review protocol. BMJ Open. (2023) 13:e071901. doi: 10.1136/bmjopen-2023-071901, PMID: 37399439 PMC10314579

[54] Ogunseitan OA. Certification in public health (CPH) Q&a Exam Review. New York, NY: Springer Publishing Company (2021).

[55] Wallerstein NB Yen IH Syme SL. Integration of social epidemiology and community-engaged interventions to improve health equity. Am J Public Health. (2011) 101:822–30. doi: 10.2105/AJPH.2008.140988, PMID: 21421960 PMC3076386

[56] Clark SE Bledsoe MC Harrison CJ. The role of social media in promoting vaccine hesitancy. Curr Opin Pediatr. (2022) 34:156–62. doi: 10.1097/MOP.0000000000001111 35232950

[57] Frohlich ED Quinlan PJ. Coronary heart disease risk factors: public impact of initial and later-announced risks. Ochsner J. (2014) 14:532–7. PMID: 25598717 PMC4295729

[58] Conway GN Han X Munro HM Gross AL Shu X-O Hargreaves MK. The obesity epidemic and rising diabetes incidence in a low-income racially diverse southern US cohort. PLoS One. (2018) 13:e0190993. doi: 10.1371/journal.pone.0190993, PMID: 29324894 PMC5764338

[59] Raghuveer G Hartz J Lubans DR Takken T Wiltz J Mietus-Snyder M. Cardiorespiratory fitness in youth: an important marker of health: a scientific statement from the American Heart Association. Circulation. (2020) 142:e101–18. doi: 10.1161/CIR.0000000000000866, PMID: 32686505 PMC7524041

[60] Besley JC Dudo A Lawrence F. Understanding scientists’ willingness to engage. Sci Commun. (2018) 40:559–90. doi: 10.1177/1075547018786561

[61] Matanoski GM. Conflicts between two cultures: implications for epidemiologic researchers in communicating with policy-makers. Am J Epidemiol. (2001) 154:S36–42. doi: 10.1093/aje/154.12.s36, PMID: 11744528

[62] Morgan-Trimmer S. Policy is political; our ideas about knowledge translation must be too. J Epidemiol Community Health. (2014) 68:1010–1. doi: 10.1136/jech-2014-203820 24942890

[63] Rychetnik L Bauman A Laws R King L Rissel C Nutbeam D. Translating research for evidence-based public health: key concepts and future directions. J Epidemiol Community Health. (2012) 66:1187–92. doi: 10.1136/jech-2011-200038, PMID: 22569750

[64] Bayer R Fairchild AL. The genesis of public health ethics. Bioethics. (2004) 18:473–92. doi: 10.1111/j.1467-8519.2004.00412.x 15580720

[65] Lee LM. Public health ethics theory: review and path to convergence. J Law Med Ethics. (2012) 40:85–98. doi: 10.1111/j.1748-720X.2012.00648.x, PMID: 22458465

[66] Childress JF Faden RR Gaare RD Gostin LO Kahn J Bonnie RJ. Public health ethics: mapping the terrain. J Law Med Ethics. (2002) 30:170–8. doi: 10.1111/j.1748-720x.2002.tb00384.x, PMID: 12066595

[67] Dawson A Jennings B. The place of solidarity in public health ethics. Public Health Rev. (2012) 34:1–15. doi: 10.1007/BF0339165626236074

[68] Levin J. Engaging the faith community for public health advocacy: an agenda for the Surgeon General. J Relig Health. (2013) 52:368–85. doi: 10.1007/s10943-013-9699-9 23519766

[69] Hu J Wang Y. The clinical characteristics and risk factors of severe COVID-19. Gerontology. (2021) 67:255–66. doi: 10.1159/000513400, PMID: 33406518 PMC7900480

[70] Martínez-Alés G Keyes K. Invited commentary: modern epidemiology confronts COVID-19—reflections from psychiatric epidemiology. Am J Epidemiol. (2023) 192:856–60. doi: 10.1093/aje/kwad045 36843016

[71] Dimitris MC Galea S Marcus JL Pan A Sander B Platt RW. What has the pandemic revealed about the shortcomings of modern epidemiology?: what can we fix or do better? Am J Epidemiol. (2022) 191:980–6. doi: 10.1093/aje/kwac012 35081616

[72] Johnson-Agbakwu CE Ali NS Oxford CM Wingo S Manin E Coonrod DV. Racism, COVID-19, and health inequity in the USA: a call to action. J Racial Ethn Health Disparities. (2022) 9:52–8. doi: 10.1007/s40615-020-00928-y, PMID: 33197038 PMC7668281

[73] Hanage WP Testa C Chen JT Davis L Pechter E Seminario P. COVID-19: US federal accountability for entry, spread, and inequities—lessons for the future. Eur J Epidemiol. (2020) 35:995–1006. doi: 10.1007/s10654-020-00689-2, PMID: 33136249 PMC7604229

[74] Saha K. Doctor-patient relationship and public trust in health science in post-COVID world: lessons from USA and India. Med Res Arch. (2021) 9:8. doi: 10.18103/mra.v9i8.2509

[75] Bowling CJ Fisk JM Morris JC. Seeking patterns in chaos: transactional federalism in the Trump Administration’s response to the COVID-19 pandemic. Am Rev Public Adm. (2020) 50:512–8. doi: 10.1177/0275074020941686

[76] Quirk PJ. Industry influence in federal regulatory agencies. Princeton, NJ: Princeton University Press (1981).

[77] Naeem SB Bhatti R Khan A. An exploration of how fake news is taking over social media and putting public health at risk. Health Inf Libr J. (2021) 38:143–9. doi: 10.1111/hir.12320, PMID: 32657000 PMC7404621

[78] Gunter J. Medical misinformation and the internet: a call to arms. Lancet. (2019) 393:2294–5. doi: 10.1016/S0140-6736(19)31206-1 31180024

[79] Dyrbye LN Shanafelt TD. Physician burnout: a potential threat to successful health care reform. JAMA. (2011) 305:2009–10. doi: 10.1001/jama.2011.652, PMID: 21586718

[80] Riegelman RK Povar GJ Ott JE. Medical students' skills, attitudes, and behavior needed for literature reading. J Med Educ. (1983) 58:411–7. doi: 10.1097/00001888-198305000-00007 6854598

[81] Morabia A. “The emergence of translational epidemiology: from scientific discovery to population health impact.” Am J Epidemiol. (2011) 173:717–8. doi: 10.1093/aje/kwq449, PMID: 21303804

[82] Hiatt RA. Epidemiology: key to translational, team, and transdisciplinary science. Ann Epidemiol. (2008) 18:859–61. doi: 10.1016/j.annepidem.2008.08.006 18823793

[83] Brownson RC Samet JM Chavez GF Davies MM Galea S Hiatt RA. Charting a future for epidemiologic training. Ann Epidemiol. (2015) 25:458–65. doi: 10.1016/j.annepidem.2015.03.002, PMID: 25976024 PMC4646613

